# Assessing mental health during an extreme weather event in southern Brazil

**DOI:** 10.47626/2237-6089-2024-0926

**Published:** 2025-09-09

**Authors:** Santiago Madeira Diefenthaeler, Alice Cacilhas, Marina Luiza Hartmann, Daniel Prates-Baldez, Simone Hauck

**Affiliations:** 1 Universidade Federal do Rio Grande do Sul Porto Alegre RS Brazil Universidade Federal do Rio Grande do Sul (UFRGS), Porto Alegre, RS, Brazil.; 2 Fundação Universitária Mário Martins Porto Alegre RS Brazil Fundação Universitária Mário Martins (FUMM), Porto Alegre, RS, Brazil.; 3 Hospital de Clínicas de Porto Alegre Porto Alegre RS Brazil Hospital de Clínicas de Porto Alegre (HCPA), Porto Alegre, RS, Brazil.; 4 Universidade Federal do Rio Grande Rio Grande RS Brazil Universidade Federal do Rio Grande (FURG), Rio Grande, RS, Brazil.

**Keywords:** Acute stress disorder, floods, psychological support, community resilience, real-time survey, mental health

## Abstract

**Objective:**

This study aimed to evaluate mental health factors in individuals affected by the floods in Rio Grande do Sul from May 10 to June 6, 2024.

**Methods:**

A real-time survey was conducted with 1,552 participants. The Patient Health Questionnaire-9 (PHQ-9), Generalized Anxiety Disorder-7 (GAD-7), and Acute Stress Disorder (ASD) symptom checklist were used to assess mental health outcomes. Sociodemographic factors, trauma exposure, rescue participation, and psychological support were evaluated. Statistical analyses included t-tests, ANOVA, linear regression, and MANCOVA.

**Results:**

High rates of depressive and anxious symptoms were found, with over half of the individuals with moderate to severe symptoms reporting no prior history of these conditions. Participants directly affected by the floods, and those with close ones affected, had higher PHQ-9, GAD-7, and ASD scores compared to the unaffected group. Moreover, younger age, female sex, lower family income, participation in rescues, and unmet psychological support needs were correlated with worse mental health outcomes. Individuals who participated in rescues had significantly higher rates of suicide ideation. Notably, psychological support had the largest effect size in mitigating mental health symptoms, regardless of trauma exposure status, when controlled for age, sex, and income, according to MANCOVA.

**Conclusion:**

The findings highlight the critical role of psychological support in disaster response and the importance of community resilience. Real-time data collection during crises can inform targeted interventions, emphasizing the need for robust mental health services and community support networks. These efforts are essential to reduce long-term psychological morbidity and foster recovery in vulnerable populations.

## Introduction

Floods are a frequent global natural hazard, with their incidence and impact increasing worldwide. Studies from the World Health Organization (WHO) and other sources confirm that floods are the most common type of natural disaster, and their frequency and intensity have been rising due to climate change.^1,2^

From late April to early June 2024, Rio Grande do Sul, Brazil's southernmost state, experienced the worst climatic disaster in the state's history.^3^ Of the 496 cities, 476 were affected, destroying houses, roads, and bridges, and isolating several towns. Government data indicates 2,398,255 people were directly affected (22% of the state's population), 581,643 were displaced, 80,826 were sheltered, and 82,666 people and 12,503 animals were rescued.^3^ Following the floods, residents faced the daunting task of returning to their submerged homes and dealing with significant losses.

Addressing post-catastrophe mental health issues is crucial, especially in countries where infrastructure is already strained. A meta-analysis shows that the prevalence of mental health disorders varies widely based on the context of the event: anxiety rates from 2.2% to 84%, depression from 3.23% to 52.7%, and Post-Traumatic Stress Disorder (PTSD) from 2.6% to 52%.^4^ While many individuals improve over time,^1^ persistent symptoms can occur. For instance, a study on a flooding event in England found that probable depression and PTSD were significantly more likely in the flooded population compared to the unaffected group even three years later.^2^

Managing resources with consideration of individual and cultural differences enhances psychological responses and builds long-term resilience, reducing future impacts.^2,5^ Understanding immediate mental health impacts and providing timely interventions can reduce long-term distress. Despite growing concern over extreme weather events, there are still gaps in the literature regarding risk factors, exposure measurements, and evaluation during and immediately after these events, limiting both understanding and the development of interventions.

This study aims to perform a real-time assessment of common mental health issues using validated screening measures during a massive extreme climate event in Rio Grande do Sul between May 10th and June 6th, 2024.

## Method

### Design, setting, and participants

This online, cross-sectional survey aimed to assess the real-time impact of the May 2024 climate catastrophe in southern Brazil. Conducted from May 10 to June 6, 2024, with ethical approval from the Hospital de Clínicas de Porto Alegre (CAAE 80017124.7.0000.5327), the study used snowball sampling to target residents of Rio Grande do Sul affected by the flood. A dedicated research email, along with Facebook and Instagram accounts, was created to facilitate participation via an anonymous online questionnaire consisting of 53 questions administered through the SurveyMonkey platform (www.surveymonkey.com). The survey was disseminated through social media channels and promoted with paid advertisements on Facebook and Instagram. No compensation was offered for participation.

While online surveys can introduce limitations such as selection bias, the decision to use a web-based platform was due to limited access to many regions of the state at the time, lack of funding, communication challenges, and the need to rapidly collect data to evaluate the mental health impact of the flood. Notably, participants had the option to leave an email at the end of the survey, and many are currently being followed to better understand the long-term mental health effects. Additionally, the results have been widely disseminated through various media outlets, including television, radio, and newspapers, with the aim of enhancing community resilience. Informed consent was obtained online, in accordance with the Declaration of Helsinki.

**Figure 1:**
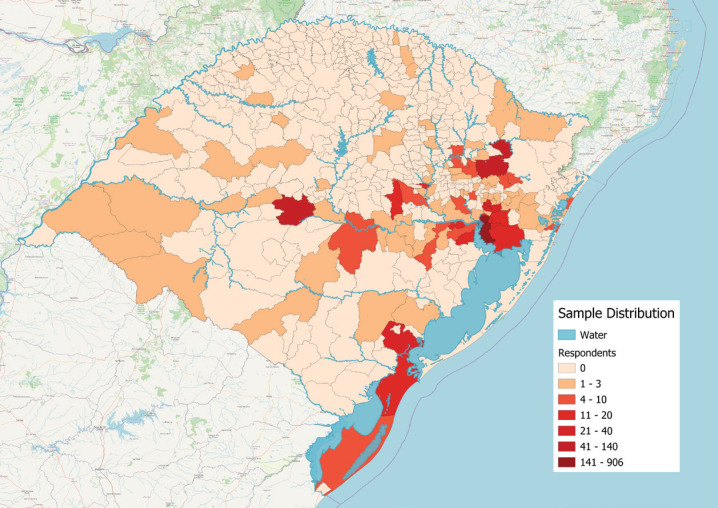
Sample distribution by cities from May 10 to June 6, 2024 (n = 1,552).

**Figure 2:**
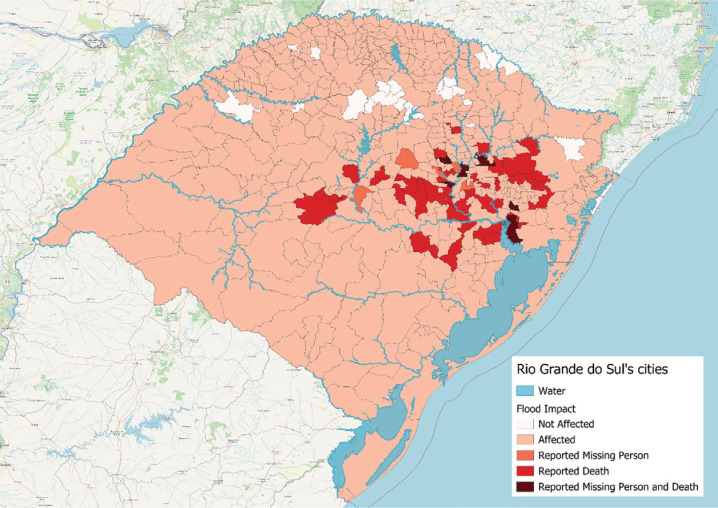
Cities affected by the floods in the State of Rio Grande do Sul.

Georeferenced mapping (Figure 1) confirmed that respondents’ residences were primarily located in flood-impacted areas (Figure 2). Inclusion criteria were survey responses within the specified dates and completion of at least one standardized instrument, while exclusion criteria were non-residency in Rio Grande do Sul and being under 18. May 6 was marked as "day one" since it was the day the central areas of Porto Alegre, the state capital, were flooded. Although water levels in central Porto Alegre began to recede around May 20, the Guaíba River only dropped below flooding levels on June 1. The Guaíba plays a crucial role in the region's flooding dynamics, as it serves as the main drainage basin for several rivers that converge in the metropolitan area. When water levels in the Guaíba rise, they can cause backflow into smaller rivers and streams, exacerbating flooding in low-lying areas. The prolonged elevation of the river's water level delayed the recession of floodwaters, further impacting affected communities.^3^

### Online questionnaire

#### Demographic and individual variables

The online questionnaire collected demographic information, including sex, age, ethnicity/race, educational level, family income, presence of children, and the city and neighborhood of residence before the flood.

#### Impact of the floods, rescue efforts, and support network

Participants were asked if they were directly affected by the flood, affected by proxy, or not affected. Specific impacts assessed included news exposure, loss of water, electricity, or internet, loss of supplies or housing access, contact with floodwater, housing loss, loss of contact with family or pets, death of a close person, transportation and workplace disruptions, direct rescue efforts participation, urgent evacuation, rescue needs, support network perception, and temporary shelter arrangements.

#### Patient health questionnaire-9 (PHQ-9)

The PHQ-9 is a brief self-administered tool designed to measure the severity of depressive symptoms. It comprises nine questions, with responses ranging on a scale from 0 to 3. The cumulative score can range from 0 to 27. The PHQ-9 is known for its strong reliability and validity.^6^

#### Generalized anxiety disorder-7 (GAD-7)

The GAD-7 is a concise self-report instrument aimed at evaluating the severity of anxiety symptoms. It includes seven items, with response options scored from 0 to 3. The total score spans from 0 to 21. The GAD-7 has demonstrated excellent reliability and validity in both clinical and research contexts.^7,8^

#### Acute stress disorder (ASD) symptoms evaluation

ASD features symptoms similar to PTSD, but they appear immediately after a traumatic event and last from three days to one month.^9^ Symptoms are categorized into intrusion, negative mood, dissociative, avoidance, and arousal, aiming to understand immediate trauma responses. In this study, 953 participants responded "yes" or "no" to each of the 14 ASD symptoms listed in the DSM-5. The remaining 599 participants used a five-point Likert scale, from "not at all" to "extremely," to assess the intensity of each symptom category.

#### Previous symptoms of depression, anxiety, and ASD

After completing each of the questionnaires, participants indicated whether the symptoms were present before the floods. The options were: "They were not present"; "They were present and remained the same"; and "They were present but worsened or increased after the event." This approach helped identify symptoms triggered by the flood and explore the relationship between previous depression/anxiety symptoms and current depression, anxiety, and ASD symptoms.

#### Suicidal ideation

Assessed via a direct question — "Since the event, have you ever thought about or wished to take your own life?" — and the 9th question of the PHQ-9.^6^ "No" for the direct question and "Not at all" for the PHQ-9 9th question were considered negative for suicidal ideation.

#### Psychological support during/after the event

Participants were asked the following question: ‘After the event, did you receive any psychological or psychiatric support or care?’ The response options were: ‘No, and I don't think I needed it’; ‘No, but I think I needed it’; and ‘Yes’.

### Statistical analysis

Descriptive analyses were conducted on all demographic and clinical variables. Means and standard deviations were calculated for numerical variables, while absolute and relative frequencies were computed for categorical variables. Normality assumption for numerical variables was assessed via visual inspection using histograms. For numerical variables, independent two-sample t-tests were conducted to compare mean scores between participants based on all relevant variables. For each t-test, Levene's test for equality of variances was performed to assess the assumption of homogeneity of variances, and the t-test results were interpreted accordingly. Significant differences in mean scores between the groups were reported, with effect sizes calculated using Cohen's d to quantify the magnitude of these differences. Welch's and regular ANOVA tests were used based on homogeneity of variances assumption. The Games-Howell test was used as post-hoc, when applicable.

Pearson correlation was conducted to assess the strength and direction of correlations between numerical variables. For categorical variables, the chi-square (χ²) test was used. The chi-square (χ²) test was employed to assess the association between ASD symptom dimensions and previous depressive/anxiety symptomatology, as well as to assess the association of involvement in rescue operations with suicide ideation and unmet need for psychological support. Effect size was evaluated using odds ratio. Fisher's exact test was also employed to confirm the significance of the association, given the small sample size in some cells.

Linear regression analyses were conducted to examine the effects of sociodemographic variables, trauma exposure, and psychological support on depressive symptoms (PHQ-9), anxiety symptoms (GAD-7), and ASD. Independent variables for each outcome included age, gender, income, and the need for psychological support, along with other variables found to be significant in previous analyses. Categorical dichotomous variables were coded as 0 and 1, with ‘no need for support’ and ‘receiving support when needed’ grouped versus ‘unmet need for support. Similarly, being directly affected or affected by proxy was grouped into one category for interpretability.

Multivariate Analysis of Covariance (MANCOVA) with bootstrap resampling was performed to investigate the effects of being affected by the flood (categorized as directly affected, affected by proxy, or not affected at all) and the need for mental health support (categorized as "No, and I don't think I needed it"; "No, but I think I needed it"; and "Yes") on three mental health outcomes: ASD, depression (PHQ-9), and anxiety (GAD-7). The analysis controlled for age, income, and sex as covariates. Moreover, 572 participants were included in this analysis, as a full factorial model with Type III sum of squares was used. We included two fixed factors: "Affected by the flood" (3 levels) and "Need for mental health support" (3 levels). By examining these factors simultaneously, we can assess not only their individual impacts on the dependent variables (ASD, PHQ-9, GAD-7) but also their interaction effects. Also, we chose Pillai's Trace as the multivariate test statistic for reporting our results due to its robustness against violations of assumptions such as homogeneity of variance-covariance matrices and multivariate normality. The effect size was measured by Eta squared (η²).

All statistical analyses were conducted using IBM SPSS Statistics Faculty Pack 29 and the R programming language. The significance level was set at p < 0.05. Figures 1 and 2 were made using the QGIS software version 3.38.0. Data for Figure 2 was retrieved from the states’ Civil Defence data for the 29th of May 2024.

## Results

A total of 1,552 individuals met the inclusion criteria. The number of individuals in each analysis varied based on the number of participants who answered each question, as many were not mandatory, and some were added after May 18, being asked only to the last 599 individuals. This adjustment was made as the study progressed and insights were gained. Among the 1,552 participants, 124 (8%) reported not being affected by the floods, 756 (48.7%) had someone close who was affected, and 672 (43.3%) were directly affected by the floods.

The majority of the sample was female (79.1%), white (88.7%), and had children (59.6%). More than half were from the capital, Porto Alegre (58.7%), with the second-largest group from Canoas (9.4%), though several other cities in the state were represented (Figure 1). The sample generally represented areas affected by the floods between late April and June 6th, including those reporting more missing people and confirmed deaths by that time (Figure 2). Further demographic data are described in Table 1.

**Table 1:** Demographic characteristics of the study participants

Variable		mean (SD)
Age		44.73 (±14.18)
Days since May 6*		14.63 (±10.5)
	
Family income		**n (%)**
	Up to 3,000 BRL	293 (19)
	From 3,000 BRL up to 10,000 BRL	672 (43.6)
	Over 10,000 BRL	575 (37.3)
	
Educational level		
	Illiterate	5 (0.3)
	Incomplete Elementary	16 (1)
	Complete Elementary	9 (0.6)
	Incomplete High School	23 (1.5)
	Complete High School	149 (9.6)
	Incomplete Higher Education	216 (13.9)
	Complete Higher Education	411 (26.5)
	Postgraduate	723 (46.6)

* On Monday, May 6, water began to invade neighborhoods in the center of Porto Alegre.

### Screening for depressive and anxiety symptoms

The mean PHQ-9 score was 9.64 (SD = 6.57) and the mean GAD-7 score was 9.91 (SD = 6.07). The severity of both anxiety and depressive symptoms is detailed in Table 2. Notably, over half of the individuals with moderate to severe symptoms of both anxiety and depression reported no prior history of these conditions.

**Table 2:** Depression, anxiety, and acute stress disorder symptoms

Variable			n (%)
Depression (PHQ-9 score), n = 1503			
	None-minimal (0-4)		365 (24.3)
	Mild (5-9)		502 (33.4)
	Moderate (10-14)		278 (18.5)
	Moderately Severe (15-19)		200 (13.3)
	Severe (20-27)		158 (10.5)
	
Anxiety (GAD-7 score), n = 1474			
	Minimal anxiety (0-4)		276 (18.7)
	Mild anxiety (5-9)		531 (36)
	Moderate anxiety (10-14)		270 (18.3)
	Severe anxiety (>15)		397 (26.9)
	
Acute stress disorder dimensions, n = 599			
	Intrusion symptoms		
		Not at all	75 (12.5)
		Slightly	198 (33.1)
		Moderately	151 (25.2)
		Very	131 (21.9)
		Extremely	44 (7.3)
	
	Avoidance symptoms		
		Not at all	161 (26.9)
		Slightly	216 (36.1)
		Moderately	113 (18.9)
		Very	86 (14.4)
		Extremely	23 (3.8)
	
	Arousal symptoms		
		Not at all	92 (15.4)
		Slightly	212 (35.4)
		Moderately	117 (19.5)
		Very	130 (21.7)
		Extremely	48 (8)
	
	Negative mood		
		Not at all	275 (45.9)
		Slightly	157 (26.2)
		Moderately	87 (14.5)
		Very	62 (10.4)
		Extremely	18 (3)
	
	Dissociative symptoms		
		Not at all	141 (23.5)
		Slightly	172 (28.7)
		Moderately	104 (17.4)
		Very	120 (20)
		Extremely	62 (10.4)

* On Monday, May 6, water began to invade neighborhoods in the center of Porto Alegre.

According to Pearson correlation coefficients, there was a very strong positive correlation between PHQ-9 and GAD-7 scores (r = 0.80, p < 0.001) across the entire sample. Additionally, age was negatively associated with both PHQ-9 (r = −0.27, p < 0.001) and GAD-7 scores (r = −0.27, p < 0.001). However, the rate of suicidal thoughts ranged between 8% and 8.5%, which is similar to or even lower than rates observed in other countries and recent Brazilian studies.^10,11^

### Screening for acute stress disorder symptoms

The first part of the sample (n = 953) responded yes or no to each of the 14 ASD questions. Only 18.5% (n = 176) reported not experiencing any ASD symptoms. The most common symptoms were difficulty concentrating (39.2%), derealization (37.4%), sleeping problems (36.1%), emotional suffering when reminded of the event (29.7%), hypervigilance (25.6%), feeling irritable or about to burst (24%), distressing dreams about the event (21.5%), recurring and involuntary memories about the event (21.3%), and an inability to experience positive emotions such as happiness and satisfaction (21.1%). Except for "being unable to remember significant parts of the event," all other ASD symptoms were associated with higher anxiety (GAD-7) and depression (PHQ-9) scores (p < 0.001).

The data about 599 participants who responded to questions corresponding to the ASD categories using a five-point Likert scale are detailed in Table 2.

It is important to clarify that the analyses were conducted separately for the two subsamples: the group that responded with yes/no to the 14 ASD questions and the group that answered the ASD/PTSD categories using a five-point Likert scale. This distinction was made to accurately reflect the different data collection methods and ensure the validity of the analyses.

### Levels of depressive, anxiety, and ASD symptoms according to trauma exposure

#### Needing urgent evacuation

366 (23.6%) participants reported having to urgently evacuate. Participants who needed to evacuate had higher mean scores across all measures: GAD-7 (M = 11.78; SD = 6.10), PHQ-9 (M = 11.49; SD = 6.69), and ASD (M = 8.61; SD = 4.68), compared to those who did not need to evacuate: GAD-7 (M = 9.32; SD = 5.94), PHQ-9 (M = 9.07; SD = 6.42), and ASD (M = 7.06; SD = 4.49). The differences were statistically significant for all outcomes: GAD-7 (t_(573)_ = −6.64; p < 0.001; 95%CI [-3.19, −1.73]; Cohen's d = −0.41; 95%CI [-0.53, −0.29]), PHQ-9 (t_(572)_ = −6.00; p < 0.001; 95%CI [-3.20, −1.62]; Cohen's d = −0.37; 95%CI [-0.49, −0.25]), and ASD (t_(597)_ = −3.61; p < 0.001; 95%CI [-2.38, −0.70]; Cohen's d = −0.34; 95%CI [-0.52, −0.15]).

#### Personal participation in rescuing people and animals

Of the 599 participants who were asked whether they were directly involved in rescuing people or animals, 87 reported being involved. Higher mean scores were found in the involved participants for GAD-7 (12.19 vs. 9.35), PHQ-9 (11.31 vs. 9.31), and ASD (9.16 vs. 7.17). These differences were statistically significant, with rescue participants showing higher anxiety (t_(581)_ = −4.11; p < 0.001; Cohen's d = −0.48), depression (t_(586)_ = −2.67; p = 0.008; Cohen's d = −0.31), and acute stress symptoms (t_(597)_ = −3.79; p < 0.001; Cohen's d = −0.44). Rescue participation was associated with a 2.39 times higher likelihood of suicidal ideation (16.1% vs. 7.4%; χ²_(1)_ = 7.051, p = 0.008; 95%CI [1.24, 4.63]). It was also linked to a greater unmet need for psychological support (54% vs. 34.2%; χ²_(1)_ = 12.55, p < 0.001; OR = 2.26, 95%CI [1.43, 3.58]).

#### Report of being affected by the flood

Welch's ANOVA revealed significant differences between the 3 groups in GAD-7 (Welch's F_(2, 333)_ = 32.56; p < 0.001; ω² = 0.016) and PHQ-9 scores (Welch's F_(2, 331)_ = 22.56; p < 0.001; ω² = 0.012). Post hoc Games-Howell tests showed that both the directly affected group and the someone close affected group had significantly higher PHQ-9 scores than the not affected group (respectively MD = 4.10; SE = 0.61; p < 0.001; 95%CI [2.66, 5.55] and MD = 3.56; SE = 0.60; p < 0.001; 95%CI [2.13, 4.99]). Similarly, for GAD-7 scores, both the directly affected group and the someone close affected group had significantly higher scores than the not affected group (respectively MD = 4.23; SE = 0.54; p < 0.001; 95%CI [2.96, 5.50] and MD = 3.91; SE = 0.53; p < 0.001; 95%CI [2.66, 5.16]). However, there was no significant difference between the directly affected group and the someone close affected group in both GAD-7 and PHQ-9 scores.

The same pattern was observed for the 599 participants who answered questions regarding the intensity of ASD symptoms. Welch's ANOVA revealed significant differences (Welch's F_(2, 138)_ = 7.33; p < 0.001; ω² = 0.011). The Games-Howell test indicated that the not affected group had significantly lower scores compared to both the someone close affected group (MD = −2.17; SE = 0.70; p = 0.008; 95%CI [-3.84, - 0.50]) and the directly affected group (MD = −2.76; SE = 0.72; p < 0.001; 95%CI [-4.49, −1.04]).

### Psychological support during/after the event

**Figure 3:**
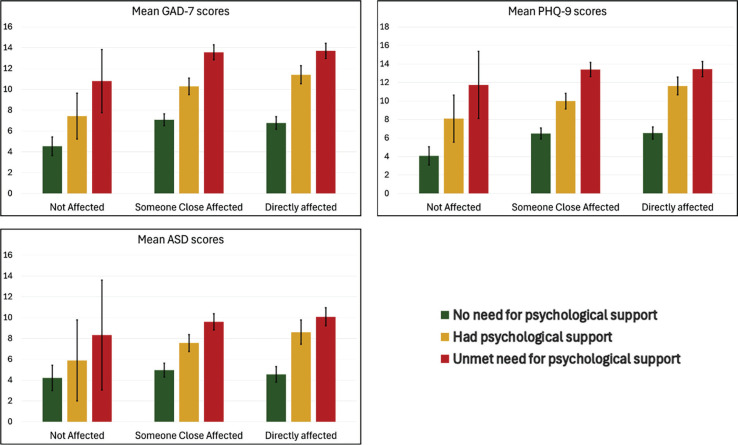
Exposure to the event and psychological support: bars represent means (95% confidence intervals).

A one-way ANOVA compared the effects of psychological support on ASD symptoms (n = 599), anxiety (n = 1,474), and depression (n = 1,503) scores among three groups: those who neither received nor felt they needed psychological support (42.3%), those who felt they needed but did not receive support (31.8%), and those who received support (26%). Significant differences were found across all groups for ASD symptoms (F_(2, 596)_ = 85.974, p < 0.001, η² = 0.224), anxiety (F_(2, 1471)_ = 228.766, p < 0.001, η² = 0.237), and depression (F_(2, 1500)_ = 212.635, p < 0.001, η² = 0.221). Welch's ANOVA confirmed these differences. Post hoc Games-Howell tests showed that those who needed but did not receive support had significantly higher scores for all outcomes, and those who received support had higher scores than those who did not feel they needed support. Figure 3 shows GAD-7, PHQ-9, and ASD mean scores according to the need/presence of psychological support after the event across each exposure level group.

### Presence of previous depressive and/or anxiety symptoms as predictors for ASD dimensions

To evaluate the impact of previous depressive and/or anxiety symptoms on ASD dimensions, we compared individuals reporting previous depressive symptoms to those without such reports. Higher frequencies were found in dissociative symptoms (60.3% vs. 49.2%; χ² = 17.05, p < 0.001), avoidance symptoms (36.4% vs. 27.9%; χ² = 11.35, p < 0.001), arousal symptoms (78.7% vs. 70.3%; χ² = 12.39, p < 0.001), and negative symptoms (42.2% vs. 27.9%; χ² = 45.75, p < 0.001). Similarly, previous anxiety symptoms were significantly associated with higher frequencies of dissociative symptoms (58.5% vs. 50.6%; χ² = 8.17, p = 0.004), avoidance symptoms (37.1% vs. 28%; χ² = 13.09, p < 0.001), arousal symptoms (78.7% vs. 70.8%; χ² = 10.56, p = 0.001), and negative symptoms (41.9% vs. 30.2%; χ² = 20.19, p < 0.001). However, previous depressive symptoms (65.2% vs. 61.6%; χ² = 1.78, p = 0.183) and previous anxiety symptoms (65.7% vs. 61.5%; χ² = 2.44, p = 0.119) were not associated with a higher frequency of intrusive symptoms. This intriguing finding warrants further exploration in future studies.

### Linear regression models considering significant sociodemographic variables, trauma exposure, and unmet need for psychological support

#### PHQ-9 (depressive symptoms)

A linear regression analysis revealed that significant sociodemographic variables, trauma exposure, and unmet need for psychological support predict PHQ-9 scores, explaining 24.3% of the variance (R² = 0.243, Adjusted R² = 0.240, F_(5, 1483)_ = 94.975, p < 0.001). The model showed that age (B = −0.098, SE = 0.011, β = −0.207, p < 0.001), female gender (B = 2.240, SE = 0.367, β = 0.139, p < 0.001), income (B = −0.993, SE = 0.211, β = -.110, p < 0.001), being affected directly or by proxy (B = 2.408, SE = 0.552, β = 0.099, p < 0.001), and the unmet need for psychological support (B = 4.446, SE = 0.329, β = 0.315, p < 0.001) significantly influence PHQ-9 scores.

#### GAD-7 (anxiety symptoms)

The linear regression model for GAD-7 scores also indicated that age, gender, income, trauma exposure, and unmet need for psychological support were significant predictors, accounting for 27.1% of the variance (R² = 0.271, Adjusted R² = 0.269, F_(5, 1455)_ = 108.405, p < 0.001). Age (B = −0.094, SE = 0.010, β = −0.215, p < 0.001), female gender (B = 2.282, SE = 0.336, β = 0.153, p < 0.001), income (B = −0.803, SE = 0.193, β = −0.096, p < 0.001), being affected directly or by proxy (B = 2.710, SE = 0.505, β = 0.121, p < 0.001), and unmet need for psychological support (B = 4.402, SE = 0.301, β = 0.338, p < 0.001) significantly predicted GAD-7 scores.

#### ASD (acute stress disorder symptoms)

For ASD symptoms, the linear regression model explained 19.8% of the variance (R² = 0.198, Adjusted R² = 0.191, F_(5, 582)_ = 28.757, p < 0.001). Significant predictors included age (B = −0.031, SE = 0.012, β = −0.093, p = 0.014), female gender (B = 1.321, SE = 0.421, β = 0.117, p = 0.002), income (B = −0.700, SE = 0.242, β = −0.111, p = 0.004), being affected directly or by proxy (B = 1.673, SE = 0.633, β = 0.099, p = 0.008), and the unmet need for psychological support (B = 3.246, SE = 0.378, β = 0.330, p < 0.001).

#### Multivariate analysis of covariance (MANCOVA)

The multivariate tests indicated significant effects of age, income, sex, and psychological support on mental health outcomes. Age showed a significant effect (Pillai's Trace = 0.020, F_(3, 558)_ = 3.883, p = 0.009), as did income (Pillai's Trace = 0.018, F_(3, 558)_ = 3.485, p = 0.016) and sex (Pillai's Trace = 0.024, F_(3, 558)_ = 4.509, p = 0.004). Psychological support had the most substantial effect (Pillai's Trace = 0.141, F_(6, 1118)_ = 14.081, p < 0.001), whereas being affected by the flood was not significant (Pillai's Trace = 0.015, F_(6, 1118)_ = 1.381, p = 0.219).

Univariate tests showed age significantly influenced ASD (F_(1, 560)_ = 5.677, p = 0.018, η² = 0.01), PHQ-9 (F_(1, 560)_ = 10.550, p = 0.001, η² = 0.018), and GAD-7 (F_(1, 560)_ = 9.770, p = 0.002, η² = 0.017) scores. Income affected ASD (F_(1, 560)_ = 9.790, p = 0.002, η² = 0.017) and PHQ-9 (F_(1, 560)_ = 4.920, p = 0.027, η² = 0.009) but not GAD-7 (F_(1, 560)_ = 2.216, p = 0.137, η² = 0.004). Sex was a predictor for ASD (F_(1, 560)_ = 9.647, p = 0.002, η² = 0.017), PHQ-9 (F_(1, 560)_ = 9.396, p = 0.002, η² = 0.017), and GAD-7 (F_(1, 560)_ = 12.108, p < 0.001, η² = 0.021). Being affected by the flood was significant only for GAD-7 (F_(2, 560)_ = 3.893, p = 0.021, η² = 0.014). Psychological support significantly reduced ASD (F_(2, 560)_ = 31.891, p < 0.001, η² = 0.102), PHQ-9 (F_(2, 560)_ = 33.813, p < 0.001, η² = 0.108), and GAD-7 (F_(2, 560)_ = 35.427, p < 0.001, η² = 0.112) scores, showing its critical role in mitigating mental health symptoms.

MANCOVA controlled for age, sex, and income, isolating the effects of being affected by the flood and psychological support. Psychological support consistently reduced symptoms across all measures, with no significant interaction effects between flood impact and psychological support.

## Discussion

The main finding of this study highlights the crucial role of psychological support in mitigating mental health symptoms during and immediately after a significant extreme weather event. Our results show that the unmet need for psychological support was the most significant determinant of PHQ-9, GAD-7, and ASD outcomes, even when controlling for sociodemographic factors and type of exposure. Individuals who did not feel the need for psychological support were the least symptomatic, suggesting greater resilience, while those who needed but did not receive support were the most symptomatic.

Significant roles of age, income, and gender were found, with younger individuals, females, and those with lower income levels being more vulnerable to anxiety, depression, and acute stress disorder symptoms, which is in line with previous findings.^12,13^ These non-modifiable factors can guide targeted interventions toward more vulnerable individuals. Pre-existing mental health symptoms were also a risk factor for more symptoms post-disaster. However, over half of those with moderate to severe anxiety and depressive symptoms after the flood had not experienced these symptoms before, demonstrating the pervasive impact of such a significant event on the community. Interestingly, although individuals with previous depressive and/or anxiety symptoms had higher prevalences of symptoms in all ASD dimensions, this was not the case for intrusive symptoms, which are central and specific to ASD and PTSD. This finding warrants further exploration in future studies at the clinical and neurobiological levels.

Also, volunteers directly participating in rescue operations had higher levels of anxiety, depression, and acute stress disorder compared to non-participants. They were also about 2.4 times more likely to experience suicidal ideation and about 2.3 times more likely to report unmet psychological support needs. These results emphasize the mental health challenges and support gaps for emergency responders, as shown in previous studies.^14,15^ Undoubtedly, first responders are a highly vulnerable population, as they are exposed to several risk factors for adverse mental health outcomes. Literature shows that repeated exposure, long periods on site, dealing with serious injuries and dead bodies, a sense of lack of control over nature, lack of training, feeling exposed to risk, and having a near-death experience are all associated with higher risk for mental disorders and tend to happen much more frequently in first responders.^16^ In this way, these populations demand better training and preparation for these situations, targeted psychological interventions, and robust support systems.^17^

However, it is crucial to clarify that symptoms of stress and anxiety during and immediately after a disaster are very common and do not necessarily indicate a mental pathology. Nevertheless, for some individuals, these symptoms may worsen or trigger a mental health condition in the long run, such as PTSD, depression, or anxiety. The literature shows that individual resilience— and even growth after a disaster—is largely dependent on the resilience of the community as a whole.^18,19^ In this context, one of the main objectives of this study was to provide data to inform the population and support the development of resilience and prevention strategies. We recognize the critical window of opportunity for prevention following a disaster, and this study was widely disseminated through various media outlets, including television, radio, and newspapers, along with a range of strategies aimed at promoting health and coping mechanisms at both individual and community levels.

Additionally, many participants voluntarily provided their contact information and are currently being followed over time. This longitudinal follow-up will allow for a deeper understanding of the trajectory of symptoms and the factors associated with more effective community responses and recovery. Our main goal with this paper was to uncover data that could support the population in rebuilding and mitigating mental health impairment. The survey remained open beyond June 6, and future analyses will enable longitudinal comparisons and tracking of symptom prevalence over time.

Although further investigation may strengthen our findings, we believe that our current analysis already provides valuable insights with great potential to assist the residents of Rio Grande do Sul in recovering from such a tragic event. Real-time knowledge of the mental health impacts of climate crises is not intended to alarm but to highlight expected reactions and encourage help-seeking for significant distress. Therefore, real-time data collection can help communities develop effective interventions by informing strategies that reduce persistent symptoms, targeting vulnerable areas, and supporting at-risk populations.

Moreover, the consistent benefits of psychological support across different levels of flood impact observed in this study underscore the need for accessible psychological services in disaster-affected areas. This highlights the importance of strengthening community support networks and disaster preparedness to ensure psychological assistance for those in need.^20–22^ Many interventions can be effectively delivered through community-level initiatives, such as psychoeducation and efforts to reduce mental health stigma, along with the provision of adequate information. These measures significantly enhance community resilience and support recovery, often without requiring specialized mental health professionals.

In fact, while most affected individuals recover, and these strategies may further increase recovery rates, a significant portion may continue to experience symptoms even years after the event.^2^ Knowledge and information about symptoms and when to seek professional help are also very important, as delaying treatment for more enduring and severe disorders can lead to more chronic and refractory conditions. For example, effective PTSD treatments exist, but only one in four individuals in low- and middle-income countries seeks help,^23^ not only due to fragile mental health infrastructures but also mainly due to a lack of knowledge about PTSD and available treatments. Psychoeducation could mitigate the event's impact in this regard as well.

An important limitation of this study is that the rates observed cannot accurately reflect the actual population due to the nature of online surveys, which have limitations such as sampling bias and self-selection bias. However, online surveys offer significant advantages, including lower costs, participant convenience, wide and fast reach, quicker data collection and analysis, and greater anonymity, which can encourage more honest responses. These characteristics make online surveys valuable for large-scale studies requiring quick responses, such as during a flood.

Moreover, collecting data during such an extreme event is unprecedented and was only possible due to the method adopted. This data is crucial and often underreported. Factors supporting the value of our data include the large number of participants, many of whom were affected by the floods, and the focus on understanding symptoms, risk and protective factors, and coping strategies, rather than exact diagnosis percentages. Some interventions can be implemented at the community level without the need for healthcare professionals. Mental health stigma and lack of information can prevent help-seeking, making studies like this and data dissemination essential. Even just the information alone can make a significant difference.

## Conclusion

The findings underscore the critical role of psychological support in alleviating symptoms of anxiety, depression, and acute stress disorder during and immediately after extreme weather events. Unmet psychological support needs are the most significant predictors of mental health outcomes, surpassing sociodemographic factors and direct exposure. Expanding mental health support networks and integrating mental health services and community resources into disaster response plans are essential. Effective interventions to reduce long-term psychological morbidity should enhance community resilience through continuous psychoeducation and stigma reduction strategies. Community-level information and interventions can make a substantial difference, emphasizing the need for robust support systems for emergency responders to ensure comprehensive mental health care during and after disasters.

## Data Availability

The data that support this study are not publicly available.
